# Nuclear DNA Sensor IFI16 as Circulating Protein in Autoimmune Diseases Is a Signal of Damage that Impairs Endothelial Cells through High-Affinity Membrane Binding

**DOI:** 10.1371/journal.pone.0063045

**Published:** 2013-05-14

**Authors:** Francesca Gugliesi, Mandar Bawadekar, Marco De Andrea, Valentina Dell’Oste, Valeria Caneparo, Angela Tincani, Marisa Gariglio, Santo Landolfo

**Affiliations:** 1 Department of Public Health and Pediatric Sciences, University of Turin, Medical School, Turin, Italy; 2 Department of Translational Medicine, University of Piemonte Orientale “Amedeo Avogadro”, Medical School, Novara, Italy; 3 Interdisciplinary Research Center of Autoimmune Diseases, Department of Translational Medicine, University of Piemonte Orientale “Amedeo Avogadro”, Medical School, Novara, Italy; 4 Rheumatology and Clinical Immunology, Spedali Civili and University of Brescia, Brescia, Italy; Carl-Gustav Carus Technical University-Dresden, Germany

## Abstract

IFI16, a nuclear pathogenic DNA sensor induced by several pro-inflammatory cytokines, is a multifaceted protein with various functions. It is also a target for autoantibodies as specific antibodies have been demonstrated in the sera of patients affected by systemic autoimmune diseases. Following transfection of virus-derived DNA, or treatment with UVB, IFI16 delocalizes from the nucleus to the cytoplasm and is then eventually released into the extracellular milieu. In this study, using an in-house capture enzyme-linked immunsorbent assay we demonstrate that significant levels of IFI16 protein can also exist as circulating form in the sera of autoimmune patients. We also show that the rIFI16 protein, when added in-vitro to endothelial cells, does not affect cell viability, but severely limits their tubulogenesis and transwell migration activities. These inhibitory effects are fully reversed in the presence of anti-IFI16 N-terminal antibodies, indicating that its extracellular activity resides within the N-terminus. It was further demonstrated that endogenous IFI16 released by apoptotic cells bind neighboring cells in a co-culture. Immunofluorescence assays revealed existence of high-affinity binding sites on the plasma membrane of endothelial cells. Free recombinant IFI16 binds these sites on HUVEC with dissociation constant of 2.7 nM, radioiodinated and unlabeled IFI16 compete for binding sites, with inhibition constant (K_i_) of 14.43 nM and half maximal inhibitory concentration (IC_50_) of 67.88 nM; these data allow us to estimate the presence of 250,000 to 450,000 specific binding sites per cell. Corroborating the results from functional assays, this binding could be completely inhibited using anti-IFI16 N-terminal antibody, but not with an antibody raised against the IFI16 C-terminal. Altogether, these data demonstrate that IFI16 may exist as circulating protein in the sera of autoimmune patients which binds endothelial cells causing damage, suggesting a new pathogenic and alarmin function through which this protein triggers the development of autoimmunity.

## Introduction

A wealth of data now exists demonstrating the critical role of interferons (IFNs) in the pathogenesis and perpetuation of autoimmunity [1]–[5]. Genomic studies have revealed that type I IFN inducible genes are markedly overexpressed in the peripheral blood of patients with systemic autoimmune diseases including Systemic Lupus Erythematosus (SLE), Systemic Sclerosis (SSc), and Sjogren’s Syndrome (SjS) [6]–[8]. In SLE patients, this so-called “IFN signature” is generally associated with active disease states, renal, and CNS involvement [9]. Together, these findings have led to the hypothesis that type I IFNs (IFN-α and IFN-β) may be the master cytokines responsible for the initiation and progression of the autoimmune process [10]–[12].

One family of IFN-inducible genes is the HIN200/Ifi200 gene family, which encodes evolutionary related human (IFI16, IFIX, MNDA, and AIM2) and murine (Ifi202a, Ifi202b, Ifi203, Ifi204, Ifi205/D3, and Ifi206) proteins. The common domain architecture of this protein family consists of one or two copies of the HIN domain (a 200 amino acid repeat) and an N-terminal PYD domain, also named PAAD, DAPIN, or Pyrin. The PYD domain, commonly found in death-family proteins, like Pyrin and ASC, is present in the N terminus of most HIN200 proteins, suggesting a role of these proteins in inflammation and apoptosis [13], [14]. The IFI16 protein is specifically expressed in vascular endothelial cells, keratinocytes, and hematopoietic cells [15] and has been recently shown to act as a foreign DNA sensor [16]–[19]. We have previously demonstrated that oxidative stress and various proinflammatory cytokines can also trigger IFI16 nuclear expression [20] and [21]. In addition, a role of IFI16 as an inducer of proinflammatory molecules (e.g. ICAM-1, RANTES, and CCL20) and apoptosis in endothelial cells has also been observed, supporting its role in the initial steps of the inflammatory processes that precede the onset of autoimmune syndromes [22]–[24]. IFI16 protein is also a target for autoantibodies. Anti-IFI16 autoantibodies have been demonstrated in the sera of patients affected by systemic autoimmune diseases including SLE, SSc, and SjS. [25]–[28]. To explain this observation, we hypothesized that its overexpression and extranuclear appearance during cell death contribute to its release into the extracellular milieu and eventually to the induction of specific autoantibodies. Consistent with this hypothesis, we have recently demonstrated *in vitro* that the IFI16 protein, normally detected in the nucleus of human keratinocytes, can be induced to appear in the cytoplasm under conditions of UV light-induced cell injury and then released in the culture media. A similar situation was also found in tissue sections of skin biopsies from patients with SLE. In this model, IFI16 expression was up-regulated and mislocalized to the cytoplasm, suggesting that aberrant expression of IFI16 in epithelial and inflammatory cells can also play a role in triggering an autoimmune response *in vivo* [29]. However, since IFI16 was previously thought to be restricted to the intracellular environment, and in particular to the nucleus [13], [30], all the recognized biological activities of IFI16, as well as their possible links with human pathologies, have only been considered in relation to this localization. Indeed, all the *in vitro* studies published to date have involved the overexpressing or down-regulation of IFI16 in different cell models, and the modulation of IFI16 has always been monitored intracellularly (i.e. using cell extracts or by directly analyzing the presence of IFI16 inside the cells, for instance using immunofluorescence techniques).

In the present study, using an in-house enzyme-linked immunosorbent assay (ELISA) we demonstrate the presence of detectable amounts of a circulating form of IFI16 in the sera from patients affected by autoimmune disorders. We also provide experimental evidence showing that the extracellular form of IFI16 is directly involved in specifically down-regulating the migratory activities and tube morphogenesis of endothelial cells. Moreover, we demonstrate the ability of IFI16 to bind to the plasma membrane of endothelial cells and, by means of binding kinetics analyses, we show for the first time, evidence of high affinity IFI16 binding sites on these cells. These data point to a new pathogenic mechanism through which IFI16 is triggering systemic autoimmune diseases.

## Materials and Methods

### Cell Cultures

Human umbilical vein endothelial cells (HUVECs), were grown in Endothelial cell growth medium-2 *(EGM-2)* (Lonza, Italy) with 2% Fetal Bovine Serum (Sigma-Aldrich, Milan, Italy) and supplemented with 1% Penicillin-Streptomycin solution (Sigma-Aldrich, Milan, Italy) as previously described [31]. Low passage human dermal fibroblasts, HDF (ATCC), mouse fibroblasts, 3T3 (ATCC), HeLa (ATCC) and HaCaT (ATCC) cells were grown in Dulbecco’s modified Eagle’s medium (*DMEM)* (Sigma-Aldrich, Milan, Italy) supplemented with 10% fetal bovine serum and 2% Penicillin-Streptomycin solution Unless specified, all cells were grown at 37°C and 5% CO_2_.

### Recombinant Proteins

The entire coding sequence of the b isoform of human IFI16 was subcloned into the pET30a expression vector (Novagen, Madison, WI) containing an N-terminal histidine tag. Protein Expression and affinity purification, followed by fast protein liquid chromatography (FPLC), were performed according to standard procedures. The purity of the proteins was assessed by 10% sodium dodecyl sulfate-polyacrylamide gel electrophoresis. As negative controls in enzyme-linked immunosorbent assays (ELISA), the polypeptide encoded by the pET30a empty vector (control peptide) was expressed and purified according to the same protocol.

### Patients and Determination of Human Extracellular IFI16 by Capture ELISA

The study groups comprised patients suffering from Systemic Sclerosis, (n = 50), Systemic Lupus Erythematosus, (n = 100), Sjogren Syndrome, (n = 49), Rheumatoid Arthritis (n = 50) and Non-SLE Glomerulonephritis (n = 46). As controls, we investigated sera from 116 sex- and age-matched healthy subjects. Written informed consent was obtained from all subjects according to the Declaration of Helsinki and approval was obtained from local ethics committees of corresponding hospital.

For the determination of circulating extracellular IFI16, a capture ELISA was employed. Briefly, polystyrene micro-well plates (Nunc-Immuno MaxiSorp; Nunc, Roskilde, Denmark) were coated with a home-made polyclonal rabbit-anti-IFI16 antibody (478–729 aa). Subsequently, plates were washed and free binding sites then saturated with PBS/0.05% Tween/3% BSA. After blocking, sera were added to plates in duplicate. Purified 6His-IFI16 protein was used as the standard and BSA served as the negative control. The samples were washed, monoclonal mouse anti-IFI16 antibody (Santa Cruz, sc-8023) added, and then incubated for 1 h at room temperature. After washing, horseradish peroxidase-conjugated anti-mouse antibody (GE Healthcare Europe GmbH, Milan, Italy) was added. Following the addition of the substrate (TMB; KPL, Gaithersburg, MD, USA), absorbance was measured at 450 nm using a microplate reader (TECAN, Mannedorf, Switzerland). Concentrations of extracellular IFI16 were determined using a standard curve for which increasing concentrations of purified 6His-IFI16 were used.

### Cell Viability Assay

Cells were seeded at a density of 1×10^4^/well in a 96-well culture plate. After 24 hours, cells were treated with different doses (10, 25 or 50 µg/ml) of recombinant IFI16 protein (IFI16), mock-treated using the same volume of vehicle as each IFI16 dose (Mock), or left untreated (NT). Where indicated, different doses (1.75 µgr or 3.5 µgr) of antibody against IFI16 were added. Forty-eight hours after treatment, cell viability was determined using the 3-(4,5-dimethylthiazol-2-yl)-2,5-diphenyltetrazolium bromide (MTT) (Sigma-Aldrich, Milan, Italy) method, as previously described [32].

### Tube Morphogenesis Assay

HUVEC were seeded in complete medium in 60-mm culture dishes coated with 0.2% gelatin (Sigma-Aldrich, Milan, Italy) and were treated for 48 h with different doses (10 or 25 µg/ml) of recombinant IFI16 protein (IFI16). As negative controls, cells were treated with the same volumes of vehicle (Mock) used for each IFI16 dose or left untreated (NT). Where indicated, different doses (1.75 µgr or 3.5 µgr) of antibody against IFI16 were added. Tube morphogenesis assay was performed as described in [33]. Briefly, a 24-microwell plate, pre-chilled at −20°C, was coated with 250 µl/well of Matrigel Basement Membrane (5 mg/ml; Becton and Dickinson, Milan, Italy) and then incubated at 37°C for 30 min until solidified. HUVEC (8×10^4^ cells/500 µl per well) were seeded onto the matrix and allowed to incubate at 37°C in 5% CO_2_. Plates were photographed after 6 h using a Leica inverted microscope.

### Migration Assay

Twenty-four well transwell inserts with an 8 µm pore size (Corning B.V. Life Sciences, Amsterdam, The Netherlands) were coated with a thin layer of gelatin (0.2%). HUVECs cultured in EGM-2 with 2% FBS and pre-treated with different concentrations of IFI16 recombinant protein or mock- or untreated for 48 hours were washed twice with PBS, trypsinized and plated into the upper chambers (4×10^5^ cells) resuspended in 200 µl of EBM-2 (Lonza, Italy), 0.1% BSA (Sigma-Aldrich, Milan, Italy) plus IFI16 recombinant protein or mock solution (the same amounts as in the 48 h pre-treatment). The lower chambers were filled with 600 µl EGM2 containing VEGF and bFGF (as chemo-attractants) (Sigma-Aldrich, Milan, Italy), 2% FBS, and IFI16 recombinant protein or mock solution (the same amounts as in the upper chamber). The chambers were incubated for 5 h at 37°C in a humidified atmosphere containing 5% CO_2_. After incubation, cells on the upper side of the filter were removed. The cells that had migrated to the lower side of the filter were washed twice with PBS, fixed with 2.5% glutaraldehyde (Sigma-Aldrich, Milan, Italy) for 20 min at room temperature, and stained with 0.5 ml crystal violet (0.1% in 20% methyl alcohol solution) (Sigma-Aldrich, Milan, Italy). After washes, color was developed in 10% acetic acid and read in duplicate at 540 nm on a microplate reader (Victor 3; Perkin-Elmer, Boston, MA).

### rIFI16-FITC Membrane Binding and Confocal Microscopy

HUVEC were seeded in 24-well plate in the presence of glass cover-slip and were grown overnight in presence of 1 µg/ml tunicamycin (Sigma-Aldrich, Milan, Italy) in EGM-2 medium with 2% FBS and antibiotics. The cells were washed twice with cold PBS and incubated with increasing concentrations (10 nM, 20 nM, 30 nM) of FITC labeled rIFI16 (FluoReporter® FITC Protein Labeling Kit by Invitrogen) for 90 minutes at 4°C. Later the cells were washed twice with cold PBS and were fixed using 2% para-formaldehyde solution for 4 minutes. The PBS wash was repeated thrice and the coverslips were mounted on glass slides using ProLong® Gold Antifade Reagent by Invitrogen. The slides were observed using Leica Confocal Microscope at 490 nm excitation wavelength for FITC in one channel while trans-illuminated light in the other.

### Co-Culturing and Immunofluorescence

Co-culturing was performed with HeLa cells and HUVEC, as described in Koristka S. et.al [34]. 10^5^ HeLa cells were seeded in 24 well-plate coated with 0.2% Gelatin in the presence of glass cover-slip and grown over-night in DMEM with 10% FCS at 37°C, 5% CO_2_. The cells were washed, suspended in PBS and lethally irradiated with UV-B lamp (HD 9021; Delta Ohm S.r.l., Padova, Italy). The dosage of 1000 Wm^2^ was counted using a UVB irradiance meter cosine corrector with spectral range of 280–319 nm (LP 9021 RAD; Delta Ohm). Followed by this, 5×10^4^ HUVEC were added in the same well, grown in EGM-2 with 2% FCS until ready. Immunofluorescence was performed after 24 hr, 36 hr and 48 hr using a home-made anti-IFI16 polyclonal as primary antibody and Alexa488- anti-rabbit (GE Healthcare) as secondary antibody. The cells were then fixed with 2% para-formaldehyde (Sigma-Aldrich, Milan, Italy), permeabilized with 0.2% Triton X-100 (Sigma-Aldrich, Milan, Italy) and nuclear stained with 1 µg/ml propidium iodide (Sigma-Aldrich, Milan, Italy). The coverslips were mounted on glass slides using ProLong® Gold Antifade Reagent by Invitrogen and the cells were observed by Leica confocal microscope.

### Radioiodination of rIFI16 and Binding Assays

Iodination Beads were purchased from Thermo Fischer Scientific Inc. (Rockford, IL, USA) and used according to manufacturer’s instructions. Briefly, two dry beads were washed with rIFI16 elution buffer (50 mM HEPES pH 7.5; 1 M NaCl) (Sigma-Aldrich, Milan, Italy), soaked dry and was incubated for 5 minutes with the solution of carrier-free 2 mCi Na^125^I (Perkin Elmer Italia, Milan, Italy) and diluted in elution buffer. Later 200 µg of rIFI16 was added and incubated for 15 minutes. The labeling reaction was passed through Zeba Spin Desalting Columns (Thermo Fischer Scientific Inc.) to remove excess Na^125^I or unincorporated ^125^I from the iodinated protein. The concentration of the final radioiodinated [^125^I]-rIFI16 was calculated using the following formula, where ‘C’ is the cpm counted, ‘V’ is volume of solution counted in ml and ‘Y’ is the specific activity of the radioligand in cpm/fmol.

Concentration of [^125^I]-rIFI16 in (pM) = [‘C’cpm/‘Y’cpm/fmol]/‘V’ml.

Binding assay was performed as described in [35], [36], 10^5^ cells/well were seeded and attached in a 24-well plate with. Once ready, the medium was removed and the cells were washed with PBS. Further they were re-suspended with increasing concentrations of [^125^I]-rIFI16 (1–32 nM) within different wells in the presence of 200 nM unlabeled rIFI16 for Non-Specific Binding. Separately, other wells were re-suspended with [^125^I]-rIFI16 (1–32 nM) without any unlabeled rIFI16 for total binding. The incubation was performed at 4°C for 90 minutes. Later, the cells were washed with PBS to remove any loosely bound ligand and were then suspended in warm 1% SDS (Sigma-Aldrich, Milan, Italy) for 5 minutes. The SDS lysate of the cells was then measured on Cobra II Series Auto-Gamma Counter. All the experiments were carried out in triplicates and the data was analyzed using non-linear regression equations from GraphPad Prism with 95% confidence intervals.

### Competition and Inhibition of [^125^I]-rIFI16 Binding

For binding competition experiments, cells were seeded into 96-well plates at a density of 10^4^ cells/well. The medium was removed and cells were washed with PBS. HUVEC were then incubated at 4°C for 90 minutes with unlabeled rIFI16 (10–1000 nM) in the presence of 10 nM [^125^I]-rIFI16. Binding inhibition was carried out overnight by incubating 10 nM [^125^I]-rIFI16 with varying concentrations (10–1000 nM) of anti-IFI16 polyclonal N-terminal (1–205 aa) or C-terminal (478–729 aa) antibodies at 4°C. This mixture was then added to 10^4^ HUVEC and incubated for 90 minutes at 4°C. The loosely bound ligand was removed by washing twice with PBS, and cells were then detached using warm 1% SDS and the levels of [125]-rIFI16-binding to HUVEC assessed using a Cobra II Series Auto-Gamma Counter. The data were analyzed using non-linear regression equations in GraphPad Prism under 95% confidence intervals.

### Statistical Analysis

All statistical tests were performed using GraphPad Prism version 5.00 for Windows (GraphPad, La Jolla, CA, USA). Positivity cut-off values for anti-IFI16 antibodies were calculated as the 95^th^ percentile for the control population and the Kruskal-Wallis test was used to measure associations. To test the effects of recombinant IFI16 protein (rIFI16) on biological functions of primary endothelial cells one-way analysis of variance (ANOVA) with Bonferroni adjustment for multiple comparisons was used.

## Results

### Serum Levels of IFI16 Protein are Increased in Patients with Systemic Autoimmune Diseases

**Figure 1:**
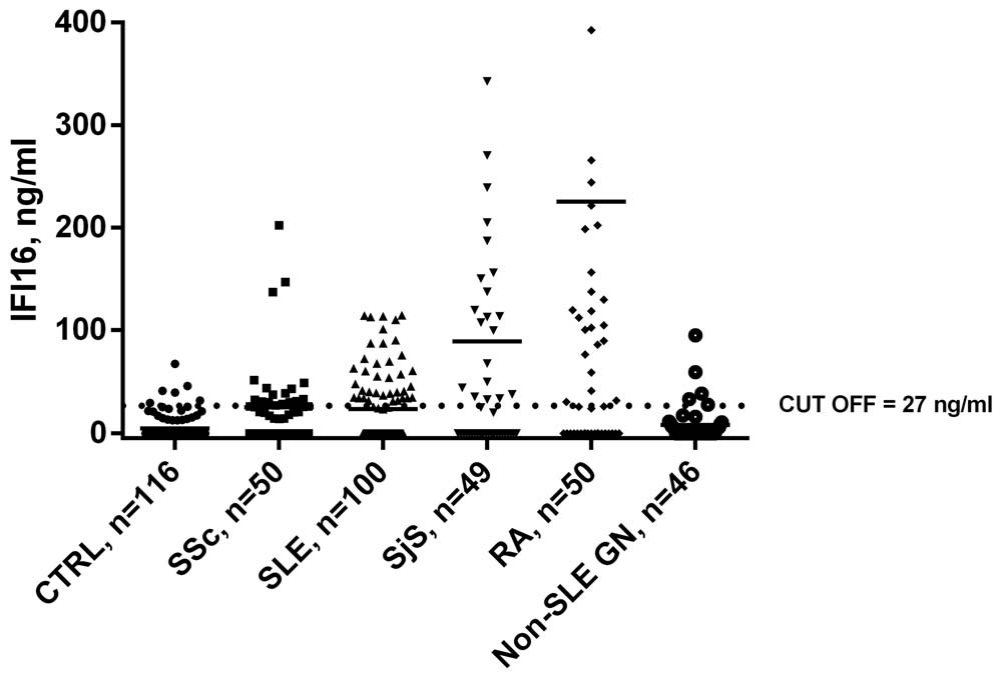
IFI16 protein levels in patients’ and controls’ sera determined using an in-house capture ELISA. Each dot represents the concentration of IFI16 protein (expressed in ng/ml on a linear scale) in each individual subject: patients suffering from Systemic Sclerosis (SSc, n = 50), Systemic Lupus Erythematosus (SLE, n = 100), Sjogren’s Syndrome (SjS, n = 49), Rheumatoid Arthritis (RA, n = 50), and non-SLE glomerulonephritis (non-SLE GN n = 46) were investigated together with healthy controls (CTRL, n = 116). The horizontal bars represent the median values. Values over the dotted line indicate the percentage of subjects with IFI16 protein levels above the cut-off value (27 ng/ml) calculated as the 95^th^ percentile of the control population. Statistical significance: *** p<0.001 *vs*. controls (Kruskall-Wallis test).

Sera were harvested from patients suffering from systemic autoimmune diseases characterized by endothelial dysfunction, including SSc, SLE, SjS, and RA. IFI16 serum levels were quantified using an in-house sandwich ELISA and compared with age- and sex-matched healthy controls. All absorbance levels were in the range of assay linearity. With the cut-off value set to the 95^th^ percentile of the control population (27 ng/ml), mean IFI16 levels were significantly increased in patients with SSc, SLE, RA, and SjS compared to the control group (4.7 ng/ml) (SSc: 25.4 ng/ml, p<0.001; SLE: 23.5 ng/ml, p<0.001; RA: 222 ng/ml, p<0.001; SjS: 88.2 ng/ml, p<0.001). Of note, the sera from RA patients displayed the highest levels of circulating free protein. IFI16 levels above the 95^th^ percentile for control subjects were observed in 34% of SSc, 37% of SLE, 47% of SjS, and 56% of RA patients (Figure 1). By contrast, IFI16 levels in non-SLE GN patients did not show any significant difference in comparison with healthy controls. Since the objective of this part of the study was limited to demonstrate the presence of circulating IFI16 in patients’ sera for justifying the rest of the in vitro studies, correlation with clinicopathological parameters was behind the aim of these studies and was not performed.

### Effects of Extracellular IFI16 on Different Functions of Primary Endothelial Cells

**Figure 2:**
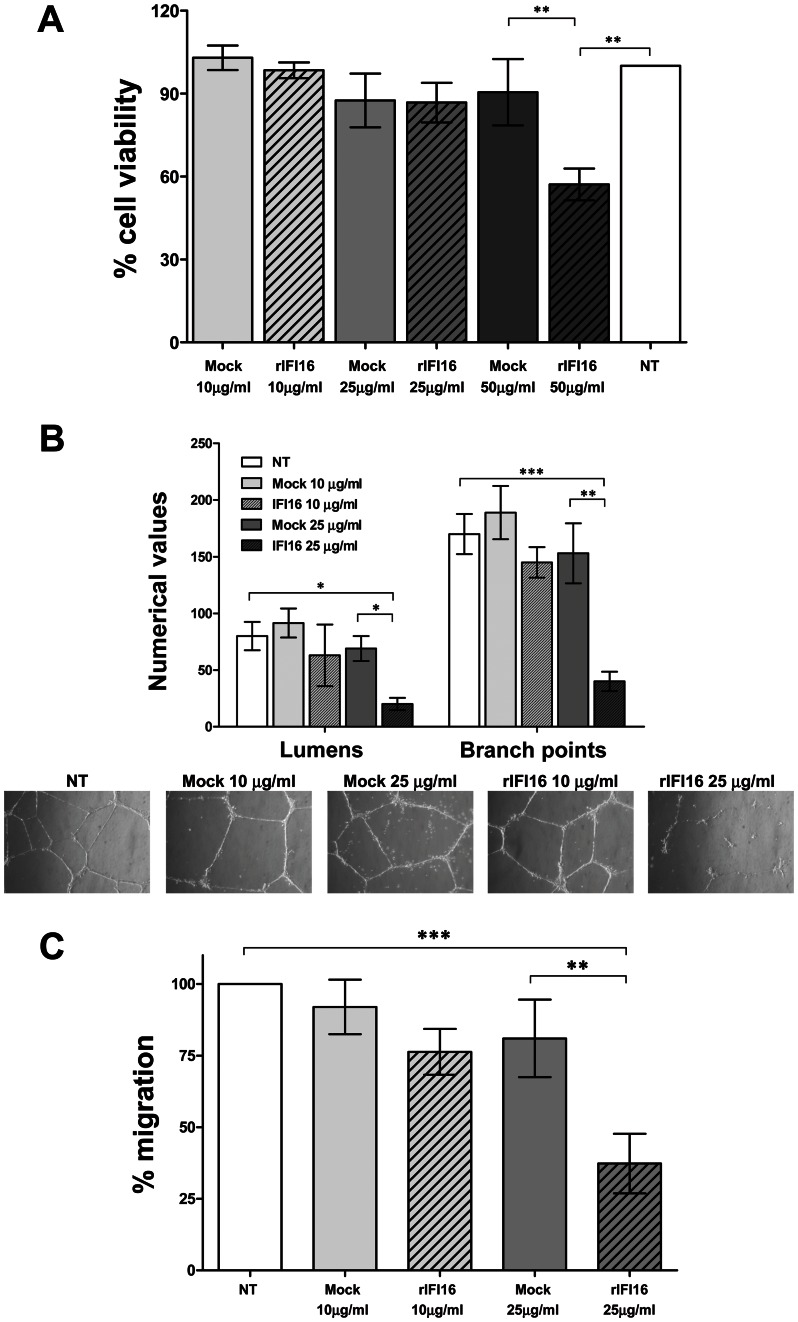
Extracellular IFI16 affects various biological functions of primary endothelial cells. HUVEC were treated with different doses of recombinant IFI16 protein (rIFI16), the same volumes of vehicle (Mock), or left untreated (NT) for 48 h. (A) Viability analysis (MTT assay); the viability of control preparations (NT) was set to 100%. (B) Capillary-like tube formation assay (Matrigel). For a quantitative assessment of angiogenesis, the number of lumens and branch points was assessed (upper panels); representative images of three independent experiments are reported (lower panels). (C) Migration analysis (Transwell assay) results are reported as the percentage of migrated cells *vs*. untreated HUVECs. Values represent the mean±SD of 3 independent experiments, (**p<0.01, ***p<0.001; one-way ANOVA followed by Bonferroni’s multiple comparison test).

Abnormalities in angiogenesis are frequently present in systemic autoimmune diseases and may lead to tissue damage and premature vascular disease [37]. To verify whether extracellular IFI16 was also involved in this pathogenic process, HUVEC were treated with increasing concentrations of recombinant IFI16 protein (rIFI16) (10, 25 or 50 µg/ml), mock-treated with the same volumes of vehicle (Mock), or left untreated (NT) and then assessed for cell viability at 48 hours incubation time by MTT assay. As shown in Figure 2A, the addition of endotoxin-free rIFI16 protein did not reduce the amount of viable adherent cells when compared to mock or untreated cells at the concentration of 10 and 25 µg/ml, respectively. At the highest concentration used (50 µg/ml), a slight reduction in cell viability was observed, and consequently the following studies were conducted with the lower doses.

Next, to test whether the addition of rIFI16 to culture media altered other biological parameters of endothelial cells, HUVEC were treated as described for the assessment of cell viability (MTT assay) and then analyzed for their tubule morphogenesis and chemotactic activities. As shown in Figure 2B, exogenous administration of 25 µg/ml rIFI16 severely limited tubulogenesis, with most cells generating incomplete tubules or aggregating into clumps. The extent of angiogenesis was quantified by counting the intact capillary-like tubules called as Lumens, which showed 75% decrease in its numerical value, as well as the number of interconnecting branch points showing 77% decrease. (Figure 2B). These effects were less pronounced when a lower dose of 10 µg/ml rIFI16 was used with 22% and 15% decrease respectively. In contrast, untreated or mock-treated HUVEC plated onto matrigel supported the formation of an extensive interconnecting polygonal capillary-like network.

Next, we evaluated the effects of rIFI16 on the migration phase of angiogenesis in a transwell migration assay routinely used to study cell migration in response to specific stimuli. HUVECs untreated, mock-treated, or incubated with rIFI16 (10 or 25 µg/ml) for 48 h were transferred into transwell migration chambers. As shown in Fig. 2C, only a small population of HUVECs cultured in the presence of 25 µg/ml rIFI16 were able to migrate through the chamber (30% of migration), whereas mock-treatment resulted in considerable migration (95% and 87% for 10 µg/ml and 25 µg/ml, respectively).

Taken together, these results demonstrate the capability of extracellular rIFI16 to impair physiological functions of endothelial cells, such as the differentiation phases responsible for tube morphogenesis and migration.

### Anti-N-terminus IFI16 Antibodies Neutralize the Cytotoxic Activity of IFI16

**Figure 3:**
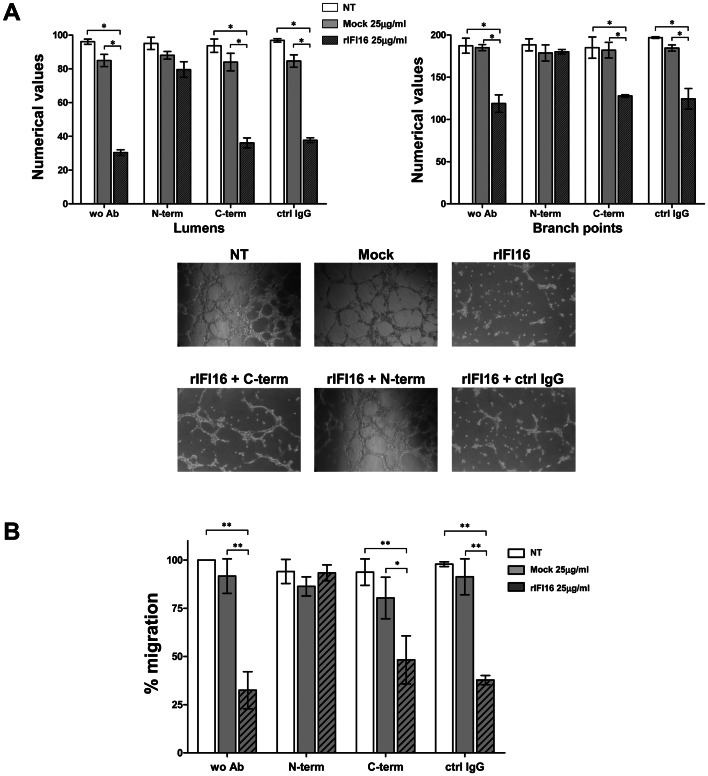
Anti-IFI16 antibodies restore the biological activities of extracellular IFI16. HUVEC were treated for 48 h with different doses of recombinant IFI16 protein (rIFI16), the same volumes of vehicle (Mock), or left untreated (NT), alone or in combination with antibodies against IFI16. (A) Capillary-like tube formation assay (Matrigel). For a quantitative assessment of angiogenesis, the number of lumens and branch points was assessed (upper panels); representative images of three independent experiments are reported (lower panels). (B) Migration analysis (Transwell assay) results are reported as the percentage of migrated cells *vs*. untreated HUVECs. Values represent the mean±SD of 3 independent experiments (**p<0.01, ***p<0.001, one-way ANOVA followed by Bonferroni’s multiple comparison test).

To demonstrate that the effects exerted by IFI16 protein on target cells were specific and not a consequence of cell cytotoxicity, HUVEC were treated with 25 µg/ml rIFI16 in the presence or absence of specific rabbit polyclonal antibodies recognizing the N-terminal or the C-terminal domain of IFI16 protein. HUVEC incubated with rIFI16 in the presence of normal rabbit IgG were used as the positive control. As described in the previous section, rIFI16 severely affected the capability of endothelial cells to generate microtubules as well as their transwell migration activity, while the same effects were observed in presence of normal rabbit IgG (Figure 3A and 3B). In contrast, the presence of anti-N-terminus antibodies reduced the Lumens by 16%, Branch Points by 5% and migration by 1% while anti-C-terminus antibodies reduced the Lumens by 60%, Branch Points by 32% and migration by 49%. This indicates the role of anti-N-terminus antibody in inhibiting the activities of IFI16 toward endothelial cells, restoring the tube formation and migratory activities. Altogether, these results suggest that the IFI16 activity is specific and that the functional domain resides at the N-terminus, where the PYD domain is localized.

### Binding of Extracellular IFI16 on the Plasma Membrane of HUVEC

**Figure 4:**
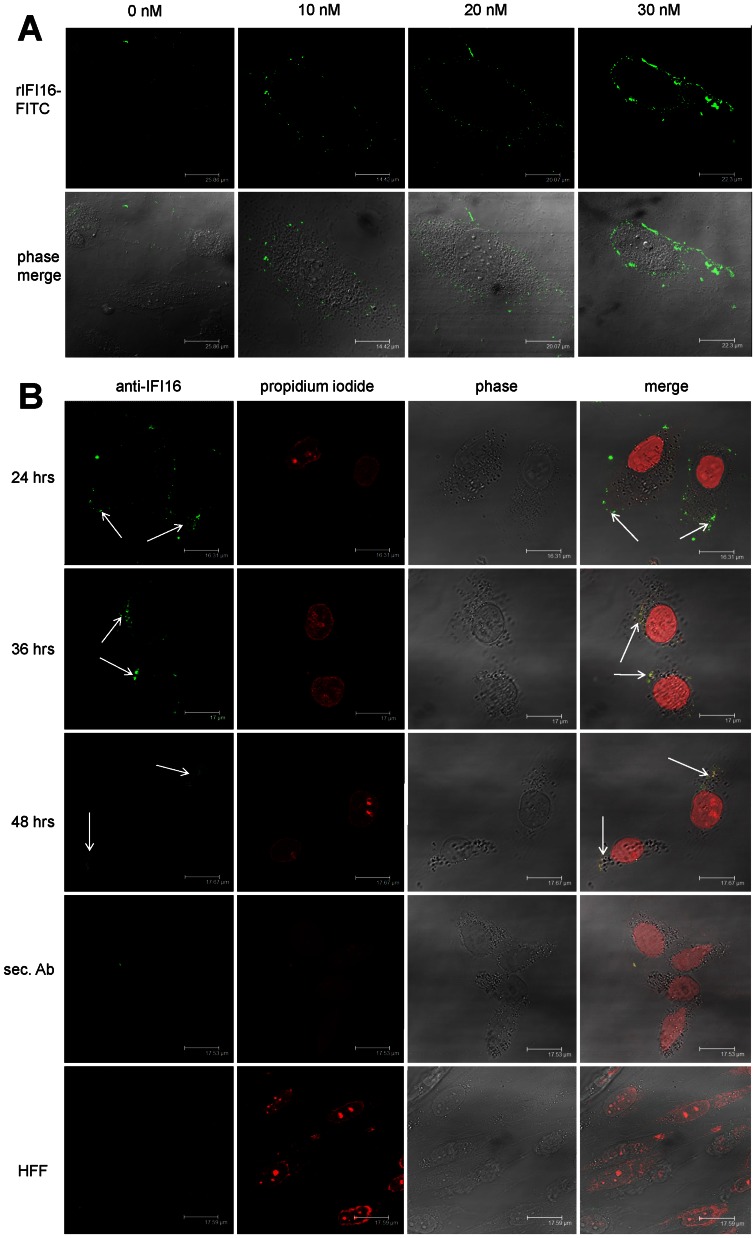
Plasma membrane binding of IFI16 to HUVEC. (A) Cells were left untreated (a, negative control) or incubated with increasing concentrations of FITC-labeled recombinant IFI16 (b, 10 nM rIFI16-FITC; c, 20 nM rIFI16-FITC; d, 30 nM rIFI16-FITC). Binding was detected by confocal microscopy using an excitation wavelength of 490 nm for FITC in one channel and trans-illuminated light in the other. Representative images of three independent experiments are shown. (B) Endogenous IFI16 released by irradiated HeLa cells binds neighboring HUVEC. UV-B irradiated HeLa cells were co-cultured with HUVEC and after 24 h, 36 h and 48 h, dead cell debris were removed and immunofluorescence was performed on the remaining live HUVEC using a home-made anti-IFI16 polyclonal as primary antibody and Alexa-488- anti-rabbit as secondary antibody. The cells were then fixed, permeabilized, nuclear-stained using propidium iodide and analyzed by confocal microscopy. Fibroblasts were employed as negative control. Representative images of three independent experiments are shown.

The finding that extracellular IFI16 impairs endothelial cell functions, including tube morphogenesis and transwell migration indicates a possible alarmin function as recently demonstrated for Danger and Pathogen-associated molecular pattern molecules collectively called as DAMPs, PAMPs such as autoantigen HMGB-1 [38], [39]. Thus to find evidence in this direction it was important to evaluate the binding interaction of IFI16 on the plasma membrane of HUVEC. A series of binding experiments were conducted to verify the presence of high-affinity binding sites in the membranes of the target cells. In the previous section, it has been described that 25 µg/ml (300 nM) rIFI16 concentration is non-toxic to HUVEC, while they can still perform biological functions. Thus HUVEC were incubated with lowest concentrations (10 nM, 20 nM, and 30 nM) of FITC-labeled rIFI16 to avoid toxicity or apoptosis and the binding was visualized by confocal microscopy. As shown in Figure 4A, binding of FITC-labeled rIFI16 was detected at least concentration of 10 nM, increased at 20 nM, and saturated at 30 nM. To avoid the non-specific binding of rIFI16 with sugar residues on plasma membrane, HUVEC were grown in presence of tunicamycin which inhibits N-glycosylation of proteins. By contrast to above findings, human fibroblasts were negative for FITC-labeled rIFI16 at all the rIFI16 concentrations investigated (data not shown).

Furthermore, to demonstrate that the binding of rIFI16 is of physiological relevance, co-culturing experiments were organized in such a way that UV-B irradiated cells release endogenous IFI16 [29] which in turn binds to neighboring HUVEC in the same system. As shown in Figure 4B, after 24 h HUVEC were observed to be surface bound with endogenous IFI16 released from HeLa cells, while by 36 h this bound IFI16 entered the cytoplasm and by 48 h it almost disappeared. When fibroblasts were used instead of HUVEC, the binding was not observed, while also when HUVEC were cultured with normal HeLa cells, surface presence of IFI16 was not detected (data not shown).

### Kinetics of rIFI16 Binding on Different Cell Lines

**Figure 5:**
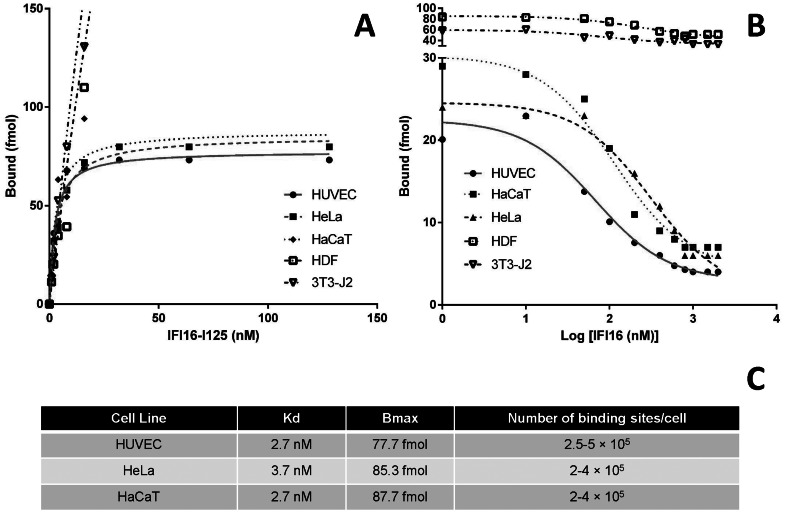
Binding Kinetics of [^125^I]-rIFI16 on HUVEC, HDF, and 3T3 cells. (A) Specific binding of [^125^I]-rIFI16 on the plasma membrane of HUVEC, HeLa, HACAT, HDF, and 3T3 cells. (B) Competitive binding of [^125^I]-rIFI16 on HUVEC, HeLa, HaCaT, HDF, and 3T3 cells. (C) The binding affinity (Kd), total bound ligand (B_max_) and the estimated number of binding sites per cell for different cell lines. The experiment was carried out in triplicates and data was analyzed using non-linear regression equations from GraphPad Prism with 95% confidence intervals. All the experiments have been repeated at least three times and one representative is reported.

To get some insights into the binding characteristics of IFI16 to different cell lines, binding kinetics experiments using radioiodinated rIFI16 were performed. Specific binding was calculated as the difference between total and non-specific binding. As shown in Figure 5A, the specific binding of [^125^I]-rIFI16 to its binding site on HUVEC is saturable and has a dissociation constant (Kd) equal to 2.7 nM; 71.55 to 83.84 fmol of [^125^I]-rIFI16 was estimated to saturate the binding sites on 10^5^ HUVEC, thus the maximal number of binding sites (B_max_) could be estimated to be in the range of 250,000 to 450,000 binding sites/cell. Furthermore, the binding of [^125^I]-rIFI16 on HUVEC was displaced by 10- to 100-fold of unlabeled rIFI16, demonstrating its competitive nature (Figure 5B). The inhibition constant (K_i_) was calculated to be 14.43 nM and the half maximal inhibitory concentration (IC_50_) was 67.88 nM. Similar results were obtained for HeLa and HaCaT cell lines, which also indicated saturable and competitive nature towards rIFI16 binding. As a negative control, human dermal fibroblasts (HDF) and murine fibroblasts (3T3) were accessed for specific and competitive binding of [^125^I]-rIFI16 in parallel with HUVEC (Figure 5A and 5B). Both HDF and 3T3 were found to exhibit non-saturable rIFI16 binding, indicating the lack of any specific IFI16 binding sites. Moreover, the binding of rIFI16 on these cells was non-competitive in nature (Figure 5B). Also as reported in Figure 5C, different cell lines shown variable affinities towards IFI16 binding.

### [^125^I]-rIFI16 Binding Inhibition by Anti-IFI16 Polyclonal Antibodies

**Figure 6:**
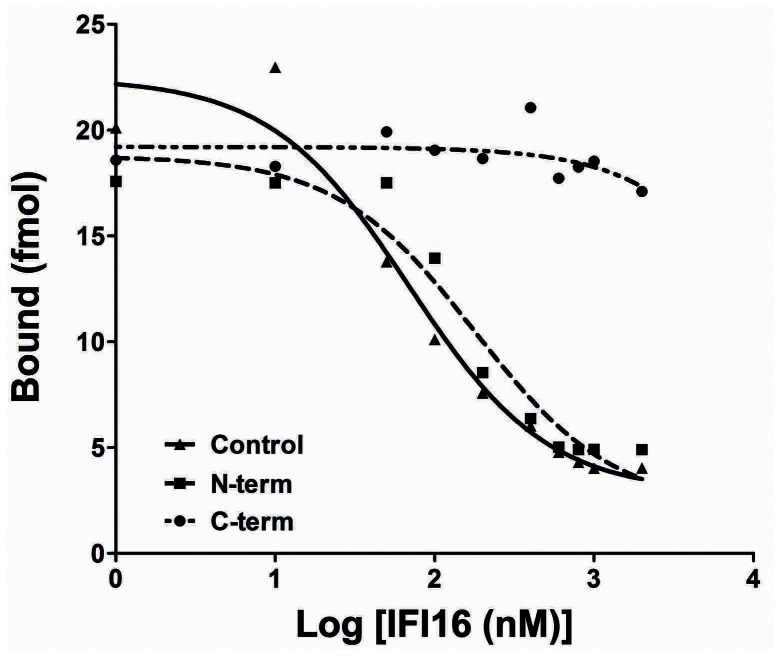
Binding inhibition of [^125^I]-rIFI16 using anti-IFI16 polyclonal antibodies. Antibody inhibition curve of HUVEC using anti-rIFI16 N-terminal (1–205 aa) polyclonal antibodies (dashed) and C-term polyclonal antibodies (dot-dashed). Competitive binding of rIFI16 and [^125^I]-rIFI16 to HUVEC (plain) was used as the control condition. The experiment was carried out in triplicates and data was analyzed using non-linear regression equations from GraphPad Prism with 95% confidence intervals. All the experiments have been repeated at least three times and one representative is reported.

To evaluate the binding properties of rIFI16 to its receptor with respect to epitope mapping, we performed a binding inhibition assay using radioiodinated rIFI16 in the presence of increasing concentrations of antibodies recognizing the IFI16 N-terminal domain. As depicted in Figure 6, a gradual decrease in the bound [^125^I]-rIFI16 was observed with increasing concentrations of antibody (from 10 to 1000 nM). Conversely, the anti-IFI16 antibody recognizing the C-terminal domain (478–729 aa) was not able to inhibit the binding of [^125^I]-rIFI16 to its receptor. Together with the results from the functional assays, these observations provide evidence indicating that the N-terminal region of rIFI16 is required for its binding to the novel membrane receptor on HUVEC and is responsible for its signal transduction capacity.

## Discussion

In the present study, we demonstrate for the first time: i) the presence of significant levels of extracellular IFI16 protein in the sera of patients affected by systemic autoimmune diseases, including SSc, SjS, SLE and RA but not in non-SLE GN as compared to healthy controls, and ii) that the extracellular IFI16 exerts biological effects on endothelial cells upon binding to a specific cell surface receptor. These findings have important implications as they provide novel insights into the role of IFI16 in the pathogenesis of systemic autoimmune diseases. Various research groups, including ours, have shown that following transfection of virus-derived DNA [19], [40], or treatment with UVB [29], IFI16 delocalizes from the nucleus to the cytoplasm and is then eventually released into the extracellular milieu. Consistent with these observations, we now demonstrate the presence of circulating IFI16 protein in the sera of patients affected by systemic autoimmune diseases, but not in patients with non-autoimmune inflammatory diseases like non-SLE GN. Skin manifestations and vasculopathy are common components of a number of autoimmune diseases and represent a significant source of morbidity [41], [42]. Thus, to investigate the hypothesis that circulating IFI16 is able to exert harmful effects on target cells *in vivo*, an *in vitro* cell model consisting of primary endothelial cells (HUVEC) was used to test the activity of extracellular IFI16 on cell functions. These experiments clearly demonstrate that extracellular IFI16 affects some biological processes of endothelial cells, including tube morphogenesis and transwell migration. The specificity of these effects was assessed by the addition of anti-IFI16 antibodies which were able to neutralize the activity of the protein blocking its inhibitory effects. Subsequently, the presence of IFI16 in the extracellular environment could also be the main reason behind the presence of anti-IFI16 autoantibodies in autoimmune patients’ sera. (Caneparo et al. Lupus 2013, accepted) Together, these observations suggest the possible role of IFI16 in the clinical manifestation of autoimmune diseases, due to its presence in the extracellular environment. Since IFI16 can be released extracellularly which further reflect distinct extracellular biological activities, it is an indication of a novel alarmin function of this interferon inducible protein. Such stress-dependent shuttling, release, binding to cell surface was described in the past for autoantigen La/SS-B [43] and recently reviewed for HMGB1 protein [39] which upon release, binds to the cell surface receptors of neighboring cells. Thus as part of alarmin function, we further hypothesized that once released IFI16 protein must also bind neighboring cells to communicate the stress signal. In this direction, we assessed the affinity of IFI16 towards the plasma membrane of HUVEC. Confocal images visualized patterned binding of FITC labeled rIFI16 protein on the plasma membrane, which gave us the first preliminary evidence of the existence of an IFI16 interacting molecule which we suspect to be receptor-kind. Furthermore, we found experimental evidence that endogenous IFI16 protein released by dying cells bind neighboring cells. As a consequence of this binding, time-lapse studies proved its further entry into the cytoplasm. Moreover, such binding and transport of IFI16 was observed in different cell lines with different affinities. The experiments using radiolabeled IFI16 to investigate the binding kinetics of IFI16 in the HUVEC provide strong evidence supporting the presence of specific binding sites in the plasma membrane through which IFI16 exerts its cytotoxic activity. These binding sites were found to be saturable and competitive for IFI16, while the binding experiments in HUVEC indicate the presence of approximately 250,000 to 450,000 binding sites per cell, with a dissociation constant (Kd) of 2.7 nM. Similar binding characteristics were shown by different epithelial cell lines while a completely un-related cell line like fibroblasts demonstrated non-specific binding. This explains the specificity of IFI16 binding which is mostly restricted towards endothelium and epithelium. Neutralization experiments employing antibodies directed against different regions of the protein allowed us to demonstrate that the N-terminus, containing the PYD domain, is responsible for binding interaction. Consistent with this observation, the same antibodies were able to neutralize the biological activity of extracellular IFI16, as described earlier.

In summary, our results provide evidence for a novel alarmin function of IFI16 protein which is overexpressed upon inflammatory stimuli and then released in the extracellular environment. Once released, IFI16 binds to neighboring cells propagating the stress signal causing damage. The presence of anti-IFI16 autoantibodies have been detected in many autoimmune diseases [25]–[28], thus the release of IFI16 in the extracellular milieu marks the first step in the development of autoimmunity.
